# Giving patients a voice: a participatory evaluation of patient engagement in Newfoundland and Labrador Health Research

**DOI:** 10.1186/s40900-020-00206-5

**Published:** 2020-07-09

**Authors:** Lidewij Eva Vat, Mike Warren, Susan Goold, Everard (Bud) Davidge, Nicole Porter, Tjerk Jan Schuitmaker-Warnaar, Jacqueline E. W. Broerse, Holly Etchegary

**Affiliations:** 1 Athena Institute, Vrije Universiteit Amsterdam, Amsterdam, The Netherlands; 2 Memorial University of Newfoundland, St. Johns, Canada; 3 NL SUPPORT Patient Advisory Council, Newfoundland and Labrador, Canada; 4 Government of Newfoundland and Labrador, St. Johns, Canada

**Keywords:** Patient engagement, Patient and public involvement, Patient participation, Monitoring, Evaluation, Outcomes, Participatory research, Participatory evaluation

## Abstract

**Background:**

Efforts to engage patients as partners in health research have grown and thereby the need for feedback and evaluation. In this pilot evaluation study, we aimed to 1) evaluate patient engagement in health research projects in Newfoundland and Labrador, Canada, and 2) learn more about how to best monitor and evaluate patient engagement. This paper presents the results of our participatory evaluation study and the lessons learned. The evaluation of the projects was driven by questions patients wanted answered.

**Methods:**

We conducted a formative evaluation of patient engagement in health research projects. Projects spanned a variety of topics, target groups, research designs and methods of patient engagement. Participants included principal investigators (*n* = 6) and their patient partners (*n* = 14). Furthermore, graduate students (*n* = 13) working on their own research projects participated. Participants completed an online survey with closed and open-ended questions about their patient engagement efforts, experiences and preliminary outcomes. Patients were involved as co-investigators in the entire evaluation study. We used qualitative methods to evaluate our participatory process.

**Results:**

The evaluation study results show that most patients and researchers felt prepared and worked together in various phases of the research process. Both groups felt that the insights and comments of patients influenced research decisions. They believed that patient engagement improved the quality and uptake of research. Students felt less prepared and were less satisfied with their patient engagement experience compared to researchers and their patient partners. Involvement of patient co-investigators in this evaluation resulted in learnings, transparency, validation of findings and increased applicability. Challenges were to select evaluation questions relevant to all stakeholders and to adapt evaluation tools to local needs.

**Conclusions:**

Our findings show that researchers, patient partners and students value patient engagement in health research. Capacity building at the supervisor level in academic institutions is needed to better support students. Sufficient time is also needed to permit observable outcomes. Participatory evaluation may increase the relevance and usefulness of information, but it also raises issues such as who defines and designs the content of evaluation tools. A co-creation process is required to develop appropriate monitoring and evaluation strategies.

## Plain language summary

“Am I making a difference?” A question asked by a Patient Advisory Council member. If we want to know how well the collaboration between researchers and patients is working, we have to ask. Patients shared questions that mattered to them. It was a challenge for the team to select evaluation questions relevant to all involved. We also struggled to find an evaluation tool that addressed all questions important to patients. If we want to evaluate the collaboration between researchers and patients, we must ensure that we ask relevant questions and adapt tools to local needs.

We adapted a tool (survey) originally developed by patients/caregivers and evaluated research projects in which patients were involved as partners. Our results show that most patients were involved in deciding what to study. They have or will be also involved in other research activities. Their insights and comments influenced research decisions. Researchers, students and patients believe their partnership can improve research. However, when patients could contribute to the research process was not clear to all patient partners. Researchers could improve the partnership by communicating more frequently during the research process. Students were less satisfied with their patient engagement experience. Training of supervisors is needed to support students. Not only research, but also its evaluation can benefit from insights from patients.

## Introduction

Patient and public engagement in health research is increasingly practiced. Growing support for patient engagement is demonstrated by its requirement from various funding agencies and medical journals [1–4]. First introduced in the United Kingdom (UK), other countries such as Canada, the United States (US), Australia, Denmark and the Netherlands also began advocating for patient engagement in research [5]. The language used to define patient engagement differs globally. There is no commonly accepted definition of ‘patient engagement’ and terms such as ‘patient and public involvement’ or ‘patient participation’ are also used. Following the Canadian Strategy for Patient Oriented Research (SPOR), we define patient engagement as ‘meaningful and active collaboration in governance, priority setting, conducting research and knowledge translation’. Patients actively contributing to research are known as ‘patient partners’, an overarching term for individuals with personal experience of a health issue and informal caregivers, including family and friends [6]. In Canada, the term patient-oriented research (POR) refers to research which engages patients alongside researchers, decision-makers and healthcare providers. This term is similar to, but distinct from, patient-centered research, participatory action research, and community-based participatory research [7].

Patient engagement is reported to improve the quality and outcomes of research, and studies show that patient engagement can impact various stages of the research cycle [8–12]. However, evaluation of patient engagement in health research remains limited. The majority of evaluation studies have been conducted by researchers in the UK and the US [13, 14]. Research funding organizations such as the Patient-Centered Research Institute (PCORI) and a Dutch research funder (ZonMw) have also conducted evaluation studies [15, 16]. In Canada, engagement initiatives are less frequently evaluated, limiting the opportunity to learn from current practices, establish best practices and demonstrate public accountability of investments. Anecdotally, patient partners, students and researchers in the province of Newfoundland and Labrador, Canada expressed the need for feedback about their patient engagement efforts; this need was the impetus for the current study.

There is no consensus on how best to evaluate patient engagement [17, 18]. We started the endeavor of co-designing an evaluation approach with patients and researchers, explicitly to pose questions relevant to them and to report results back to them to enhance their partnership. Our pilot evaluation study aimed to assess patient engagement in research projects, but at the same time provided insights into methods used for evaluation. This paper presents the results of our pilot evaluation study and our lessons learned regarding evaluation itself. We hope our experiences can inform others who wish to evaluate patient engagement.

### Context for this evaluation

The Canadian Institutes of Health Research (CIHR) announced Canada’s Strategy for Patient-Oriented Research (SPOR) in 2011 [1]. A key part of the strategy was the creation of provincial Support for People and Patient-Oriented Research and Trials (SUPPORT) Units whose mandate is to facilitate patient-engaged research on jurisdictional priorities and build capacity for patient engagement [19]. The Newfoundland and Labrador Support Unit (NL SUPPORT) offered training and research grants to build the capacity for patient-oriented research (POR). A Patient Advisory Council (PAC) was established to guide this work. The council comprises over 20 residents from around the province with varying employment, training and health backgrounds. All members are involved in research projects, governance, and/or grant review committees.

Unique to SPOR SUPPORT infrastructure is the development of a nationwide curriculum. The *Foundations in Patient-Oriented Research* course was designed and piloted in Canada to build mutually beneficial relationships for conducting patient-oriented research. Three course modules include: Module 1: Introduction to Patient-Oriented Research; Module 2: Fundamentals of Health Research in Canada; and Module 3: Building Partnerships and Consolidating Teams [20]. This curriculum has been evaluated separately [20]. In the province Newfoundland and Labrador, this curriculum was mandatory for all students who received a fellowship from NL SUPPORT and highly recommended for researchers applying for grants. Patient partners could participate on a voluntary basis; some preferred not to be trained as they felt their ‘lay’ understanding of research was important to retain. Furthermore, monthly webinars were offered open to anyone with an interest in POR. These webinars provided information on various POR topics and examples of patient engagement specific to Newfoundland and Labrador. In addition, NL SUPPORT offered training for researchers and students about ways to recruit patients as partners [21] and also provided matching services for those who did not have access to the patient community. Additional training and support was available upon request. Supporting resources such as local guidelines on research ethics and compensation of patient partners were shared with grant applicants.

A biannual competition provided a number of awards to Masters and PhD students working on their patient-oriented research thesis project. Furthermore, an annual competition provided funding to researchers to support small POR projects that are of relevance to the people of Newfoundland and Labrador. Research priorities were identified through public consultation via town hall meetings [22] and a province-wide survey and shared in the call for proposals. Proposals were reviewed by patients, researchers, health professionals and policymakers, all with an equal say in the decision-making process. Patient engagement in the research and the relevance of the proposal to research priorities were key for evaluating all funding proposals. So far, only the progress of funded projects had been monitored. By this pilot participatory evaluation study, we aimed to learn more about how to best monitor and evaluate patient engagement and its findings. This formative evaluation focuses only on patient engagement in research projects funded by NL SUPPORT. A formative and summative evaluation of the SPOR program and NL SUPPORT has been conducted separately, in which patient engagement was a component but not the explicit focus [23].

## Methodology

A participatory evaluation approach was adopted for all phases of the evaluation process. While participatory evaluation is a term widely used, other similar terms exist such as collaborative evaluation, participant-oriented approaches, responsive evaluation and/or empowerment evaluation [24–26]. The definitions and meanings of terms vary in the literature; however, the approaches show similarities to patient engagement in research. Participatory evaluation engages stakeholders in the evaluation process. Rationales include increasing the utilization of evaluation results, creating an interactive learning environment, and improving the quality of the evaluation. Evaluation becomes a team effort in which the primary investigator is also a facilitator of the evaluation process [24, 25]. In this methodology section, we first describe the evaluation design and next the participatory process of the evaluation study. Patient co-investigators were involved in the entire evaluation process, from selecting the evaluation questions, the development of the measurement tools, the interpretation of study findings up to the dissemination activities. A glossary of terms can be found in Table 1.

**Table 1:** Glossary of terms

Term	Description
Formative evaluation	Formative evaluation is typically conducted during program development or implementation in order the strengthen or improve a program [27].
Summative evaluation	Summative evaluation is typically conducted once a program is established in order to examine the effects or outcomes of a program [27].
Participatory evaluation	Participatory evaluation systematically invites and engages stakeholders in program evaluation planning and implementation [24, 25].
Statistics	Statistical methods are used for analysing and summarizing data by for example calculating the mean or standard deviation and testing the relationship between data sets (e.g. the relationship between inputs and impacts).
Descriptive qualitative approach	A research method that is used to provide a comprehensive summary of data reported in words. Data are analysed without a pre-existing (a priori) set of codes or assumptions [28].
Value model	A visual illustration that presents the expected impact of a program
Logic model	A visual illustration (road map) that presents the relationship between inputs, activities, outputs, outcomes and impacts of a program. A logic model is often used to guide evaluation planning and/or analysis.

### The evaluation study methodology

#### Setting of the study

As of 2018, NL SUPPORT funded 11 research projects with a duration of one year (with the possibility for a one-year extension which was given to two projects). The proposed patient engagement methods varied per project from an advisory capacity (e.g. consulted periodically) to patient partners as team members (e.g. attending team meetings). Patients were listed as co-applicants (part of the core research team) on most research proposals. The number of patient partners involved varied per project. Most patients involved in the projects were people with personal experience of a health issue or caregivers, while some were associated with a local patient or community organization. The projects employed qualitative, quantitative and mixed methods designs. Similarly, the projects spanned a variety of topics and target groups such as primary and tertiary care services for people with breast cancer, young adults with cancer, people with cardiovascular risks or obesity, people with mental health problems, families with autism and immigrants. Projects were mainly located in the capital city of St. John’s, with a few projects conducted in rural areas across the province. Furthermore, NL SUPPORT had funded 19 students working on their own research project with patient partners and their supervisor. As this is a formative evaluation study, we selected all student projects (*n* = 19) and the POR projects midway through their research process (*n* = 8 out of 11).

#### Evaluation tool selection and development

To avoid duplication and enable comparison across studies, the evaluation team (including researchers and patient co-investigators) first reviewed the Patient and Public Engagement Evaluation Toolkit [29] in light of the evaluation questions chosen. The tools were rated on scientific rigour, patient and public involvement in their creation, comprehensiveness and usability. Three tools with high assessment scores were selected for further review and included: Public and Patient Engagement Evaluation Tool (PPEET), Research with Patients and Public involvement (RAPPORT) and Patients as Partners in Research created by Patients Canada. A review grid was created based on the evaluation questions: attitude/expectations (Q1), supporting factors and barriers (Q2), impact on research (Q3) and uptake of research (Q4). Additional criteria included: health research focus, possibility to collect patient and researcher perspectives, possibility of using the tool for tracking over time, the evaluation method, and time investment. A full comparison of the tools can be found in Additional file 3.

The evaluation team reviewed the three selected tools. One of the key issues was that none of the tools fully captured all criteria as described above, although several did have relevant sections. The PPEET tool was suggested by one of the researchers as this tool had been validated. However, after review by the evaluation team, it was noted the tool has not been developed for a health research context and does not offer the opportunity to easily compare responses of patients and researchers. Therefore, the evaluation team agreed that the PPEET tool was not the best fit for our evaluation study. The RAPPORT study tools had most relevant sections, however a very limited quantitative component which was considered as important to easily compare responses of large groups and multiple POR projects over time. The tool (surveys) developed by patients and caregivers for Patients Canada, *Patients as Partner in Research* [30, 31], was chosen as the primary survey template as this tool met most of our criteria and offered the opportunity to compare the perspectives of researchers and patients. The option of using the tool for monitoring over time was also considered important. Furthermore, the tool was developed by patient advisors with a focus on and experience with involvement in research projects, which was considered another important advantage. Lastly, the survey method offered an opportunity to easily collect data of large groups over time while requiring limited resources.

The evaluation team slightly adapted the mid-project survey for this pilot evaluation study (e.g. a few yes/no response scales were replaced for 7-point scales, some questions were adapted to statements). Additional, qualitative, items were adapted from the RAPPORT study tools [8] to complement the closed-ended questions. Surveys were adapted for each group involved in the selected research projects (students, researchers and patient partners). The adaptation process was iterative, with drafts created by researchers on the evaluation team (LEV, HE), then sent to the patient co-investigators for in-depth review and testing. Items and response scales were deleted, added and adapted based on their feedback (e.g. questions about beliefs related to the impact of engagement on research uptake were added; some questions were deleted as the original survey was considered as too long). Principal investigator and patient partner surveys were identical except for minor wording changes relevant to each group. The surveys consisted of four parts: 1) project details; 2) participation in the project; 3) overall assessment; 4) demographics/characteristics of the respondent. The patient partner survey included six open-ended questions out of 28 questions and several comment boxes, the researcher survey included similar questions (total five open-questions out of 25). Another survey was created by the team for students and gathered information on how patients were engaged in their research, their beliefs about the value of patient engagement, as well as their experiences and challenges to date. The student survey included a few similar questions as included in the principal investigator and patient survey. The surveys can be found in additional files 4, 5 and 6. Participation was voluntary and responses were anonymous and kept confidential.

### Recruitment and data collection

All principal investigators of POR projects midway their research (*n* = 8) were invited to participate in this pilot evaluation study. They were asked to share an invitation letter with their patient partners, of which seven principal investigators did. Seventeen patient partners agreed to share their contact details with the evaluation team. In addition, 19 graduate students were invited to participate. Participants and projects names were given a de-identifiable number to make it possible to monitor over time, with only one evaluator having access to the password-protected identifiable excel file. All participants were invited via email in June 2018; this email included a unique personal link to the online survey. Participation was voluntary, and a reminder was sent after two weeks. Non-responders received a final reminder in August 2018. Six (out of 8) principal investigators, fourteen (out of 17) patient partners and thirteen (out of 19) students responded. All responses were kept confidential. Tables 2 and 3 summarizes the invited participants and responses per funding year.

**Table 2:** Study participants of NL SUPPORT patient-oriented research grants

Funding call year	Number of projects midway their research in May 2018	Invited participants	Response
2016–2017 (fall)	N = 2	N = 2 principal investigators *N* = 2 patient partners	N = 1 principal investigators *N* = 1 patient partner
2017–2018 (fall)	N = 6	*N* = 6 principal investigators *N* = 15 patient partners	N = 5 principal investigators *N* = 13 patient partners

**Table 3:** Study participants of NL SUPPORT educational funding

Funding year	Number of projects	Invited participants	Response
2016 (fall and spring)	N = 8	N = 6 MSc students N = 2 PhD students	*N* = 4 MSc students N = 1 PhD student
2017 (fall and spring)	*N* = 11	N = 11 MSc students	N = 8 MSc students

#### Data analysis

Descriptive analyses were performed using the survey platform to analyse the data collected through the survey, in particular the number of participants endorsing response options and multiple-choice questions. We (LEV, HE) examined the number of responses per respondent group (students, researchers and patient partners). Given the low numbers, we could not perform statistics on survey responses. We (LEV, HE) analyzed responses from open-ended questions using a descriptive qualitative approach [28]. Here, no a priori assumptions were made about the data. Rather, the goal was to provide a comprehensive summary of responses arising from the data.

**Fig. 1:**
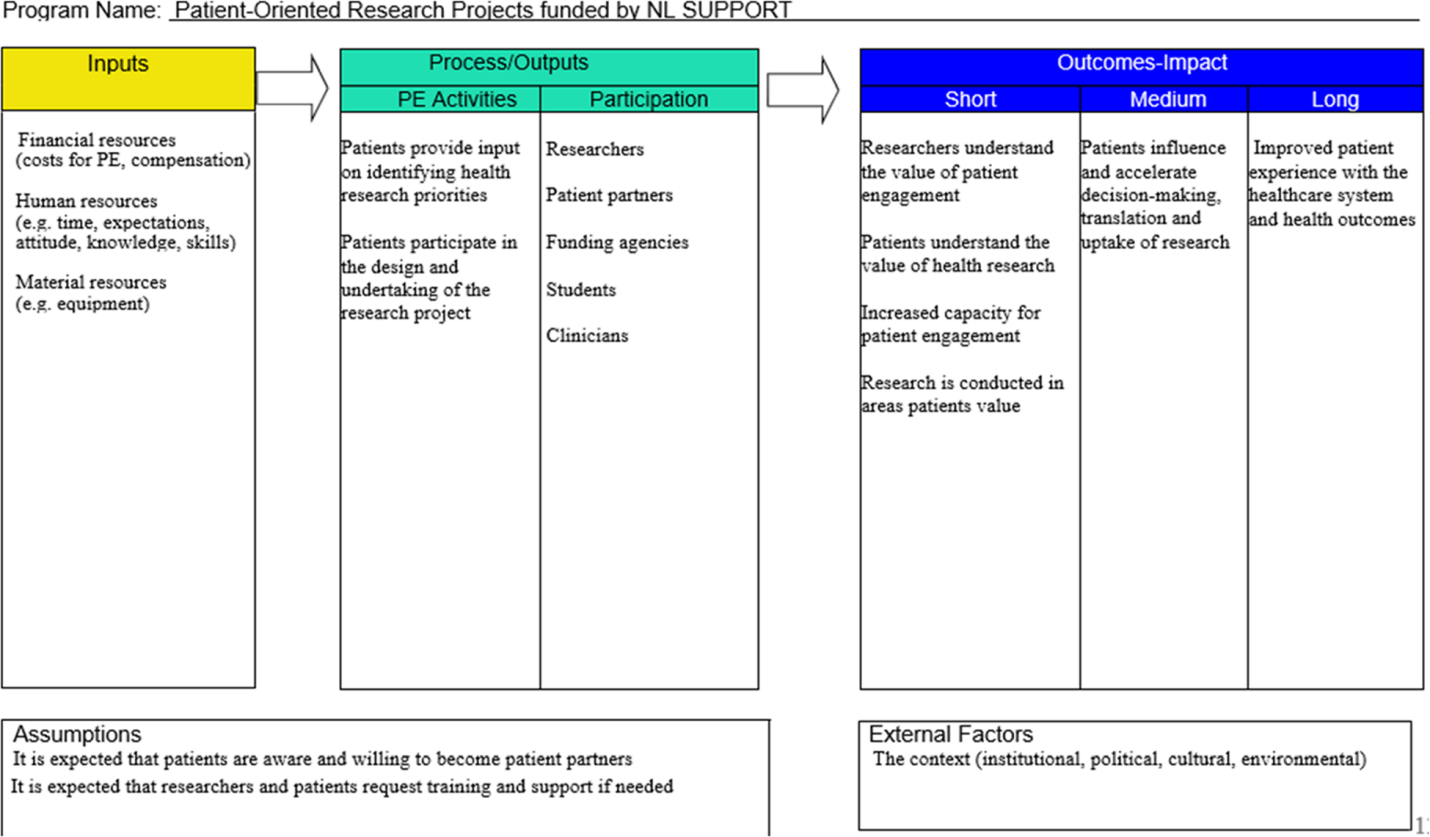
Logic model based on the SPOR Visual Value Model for Patient Engagement [6]

The SPOR program is partly based on theory explaining how patient engagement is understood to contribute to a chain of results that should lead to actual impacts. The SPOR’s National Steering Committee developed a Visual Value Model for Patient Engagement [6] which we used for the development of a logic model (see Fig. 1). We used the logic model to further categorize and report our findings.

### The participatory process methodology

#### Methods used for patient engagement in this evaluation study

Before setting up the evaluation study, we held a participatory workshop with the full patient advisory council of NL SUPPORT to identify potential evaluation questions. We asked them the question: “What would you like to learn/know about patient engagement?” Three members of the patient advisory council joined the evaluation team (BD, MW, SG). They were involved in selecting the evaluation questions, the development of the measurement tools, piloting the survey, the interpretation of study findings and dissemination activities. These patients were not involved in the research projects selected for this evaluation study. In addition, we engaged other key stakeholders such as the funding and implementing agency (NL SUPPORT), researchers and an evaluation expert (NP). The team consisted of a principal investigator (LEV) and a co-principal investigator (HE). Additional file 2 provides an overview of the stakeholders involved per phase of the study.

Through a consensus meeting, the following evaluation questions were selected:

1. What expectations and attitudes do patient partners, researchers and students have about patient engagement in health research? (Q1)
2. What supporting factors and barriers do patient partners, researchers and students experience? (Q2)
3. To what extent do patient partners, researchers and students feel that partnering with patients in health research has a meaningful impact on the research process? (Q3)
4. To what extent do patient partners influence the uptake of research? (Q4)

#### Data collection and analysis

We have used qualitative research methods such as observations, notes, recordings of meetings and written feedback to track patient inputs and our decision-making process. In the fall of 2019, we organized an online focus group with the evaluation team to reflect on our process and the impact patient engagement had on our evaluation study and the people involved in the evaluation process. The focus group was attended by all members of the evaluation team (three patient co-investigators and two researchers). The focus group was facilitated by one of the researchers (LEV). Prior to the focus group, participants received a brief recap of the evaluation study and the decisions made as well as a set of open-ended questions to prompt the discussion. We have used the GRIPP2 [32] for the development of focus group questions, which included topics such as the influence of patients on the decision-making process; positive and negative effects of engagement and recommendations.

The session was conducted and recorded using online meeting technology. One investigator (LEV) transcribed the focus group and categorized the findings into three main themes: positive outcomes and impacts, negative outcomes and impacts, process and context factors (based on GRIPP2 [32] reporting checklist – see Additional file 1). We did not make a distinction between outcomes and impacts, however we separated the effects patient engagement had on the evaluation study and the people involved. The focus group transcription and analysis was sent to all (co)-investigators for review.

## Results

The first section will present the findings of the evaluation study using the logic model to structure our findings. In the second section, we report on the evaluation process and results of patient engagement in our evaluation study.

### Findings of the evaluation study

Table 4 provides a summary of the demographics of all study participants. Below, we describe the main findings of the evaluation study. We did not observe any differences in our data based on the type of study, design nor study site or topic.

**Table 4:** Overview of the demographics of study participants

Participants	Demographics	Number (N)
Researchers (*N* = 6)	Career	Junior (*n* = 2) Mid career (*n* = 2) Senior (n = 2)
	Background	Researcher (*n* = 3) Clinical scientist (n = 3)
	Sex	Female (*n* = 5) Male (*n* = 1)
Patient partners (who worked with researchers) (*N* = 14)	Age	22–92 years, mean = 46,5 years
	Sex	Male (n = 1) Female (*n* = 13)
	Level of education	University degree (*n* = 9) Trade school or college (n = 3) High school (n = 2)
	Current work status	Working full-time (n = 8) Working part-time (n = 1) Not in labour force, unable to work (n = 1) Student (n = 2) Retired (n = 2)
Students (N = 13)	Faculty	Medicine (*n* = 7) Clinical Epidemiology (n = 2) Pharmacy (n = 2) Genetics (n = 1)
	Sex	Male (n = 5) Female (n = 7) Other (n = 1)

#### Inputs

We mainly looked at human resources, in particular the attitudes, motivation, knowledge, skills and time invested, to answer the evaluation questions 1 and 2.

All researchers indicated that they attended training sessions about patient engagement in health research. All felt that they were somewhat or well prepared to work with patient partners on the research project. They also felt that the patient partners were prepared to contribute to the research project. Five out of fourteen patients attended training sessions about patient engagement in research. All patients indicated that they felt comfortable with their understanding of the research project. Only one person did not feel equipped to contribute to the research project. All felt that the research team was (somewhat) well prepared to work with patient partners. Eight out of thirteen students somewhat to strongly agreed that they felt well prepared to work with patient partners.

Three of our six researchers indicated that their primary motivation to work with patient partners was because they felt that patients and caregivers would add value; others stated that it was required by the funder. Four of six researchers agreed or strongly agreed that patient engagement was a good use of their time and resources, while twelve of the fourteen patient partners agreed or strongly agreed. Four students felt that patients and caregivers would add value, whereas four other students said that it was not their decision to work with patient partners. The hours patient partners and researchers spent on patient engagement varied from less than 1 h a month up to more than four hours a month.

#### Process and outputs

We looked at whether or not patients provided input on identifying health research priorities and their participation in the design and undertaking of the research project as presented in the logic model. In addition, we looked at supporting factors and barriers of the engagement process (Q2).

Midway through research projects, patient partners had contributed mostly to the initial stages of the research cycle, with eleven indicating they had helped identify and prioritize the research topic. Notably, all researchers (*n* = 6) indicated patient partners had or would contribute to analysing the data and with dissemination and implementation of study findings; comparatively fewer patient partners (n = 6 out of 14) indicated involvement in these stages of the research process. Students indicated that patient partners contributed mainly to the topic identification and study design phase. One student shared:

*“When I first came onto this project, it was mostly all formed, and so I did not get much say in the way the project was designed. If I had known more about patient engagement in the beginning I would have emphasized its use in more than just the beginning and the end. I don't want to tokenistically involve patients. I want them to contribute meaningfully” (student 4)*

The most commonly reported challenge to patient engagement from a researcher perspective was the time commitment, though most recognized this was part of the process and necessary. As one researcher noted:

*“If you want to go fast, go alone. If you want to go far, go with others” (researcher, project 3)*

Another researcher stated that one of the difficulties was:

*“Shifting our thinking and experiences in academia to the everyday experiences and realities of patients” (researcher, project 4)*

Another researcher shared:

“*I learned the importance of ensuring that the patient partners have the trust in the researcher and are given the time, space and authority to speak out; they can't be timid people, and the researcher must have a lot of humility and accept being told that they have it wrong or that a certain idea or research strategy needs to be changed” (researcher, project 5)*

Patient partners experienced difficulties in communication, timing and the frequency of engagement. For example, one patient partner said:

*“I think if I had been involved from the beginning that it was really supported from what I've read and heard from the research team. I came on late and I expect that's why I feel somewhat less engaged than if I had been involved in the early part of the project. Besides attending meetings I was expecting to have take-aways to be done, which hasn't occurred at least to this point” (patient partner 3, project 1)*

Another patient partner shared:

*“More organized time table / schedule for meetings - More frequent communication on project progress - Very involved up to getting the grant approved, have not had as much insight into the project since that time” (patient partner 2, project 7)*

#### Outcomes

As the projects included in this study were midway through their research, we can only report on a few short-term outcomes and some expectations regarding medium-term outcomes as shown in the logic model. In line with our evaluation questions, we specifically focused on the influence of patients on research decision-making and the expected impact on the uptake of research (Q3 and Q4; medium-term outcomes in the logic model).

**Fig. 2:**
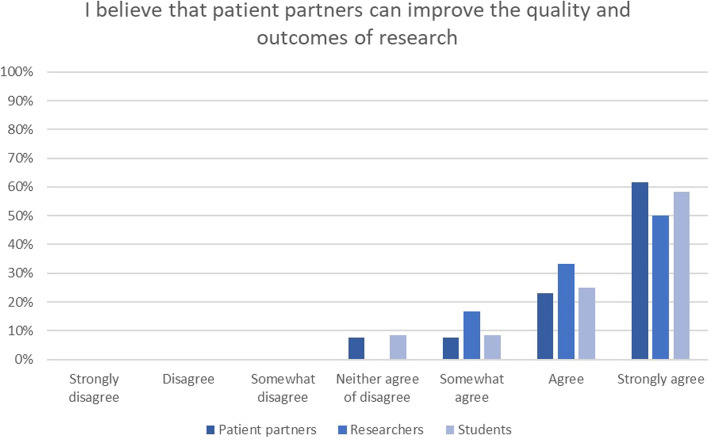
Responses to the statement ‘I believe that patient partners can improve the quality and outcomes of research’ shown per group (students, patient partners and researchers)

Figure 2 shows that most researchers, students and patients believe the partnership between patient partners and researchers can improve the quality and outcomes of research. One researcher noted:

*The assumptions of the academic researcher (me) are more easily revealed and corrected when patient partners are actively engaged and able to challenge the researcher's way of thinking about an issue - this has been really a very insightful and useful thing for my own development as a researcher, and for the quality of the data collected” (researcher 1, project 5)*

Five out of six researchers strongly agreed that patient partners can help with the translation and uptake of research, twelve of the patient partners and nine of the students agreed or strongly agreed.

**Fig. 3:**
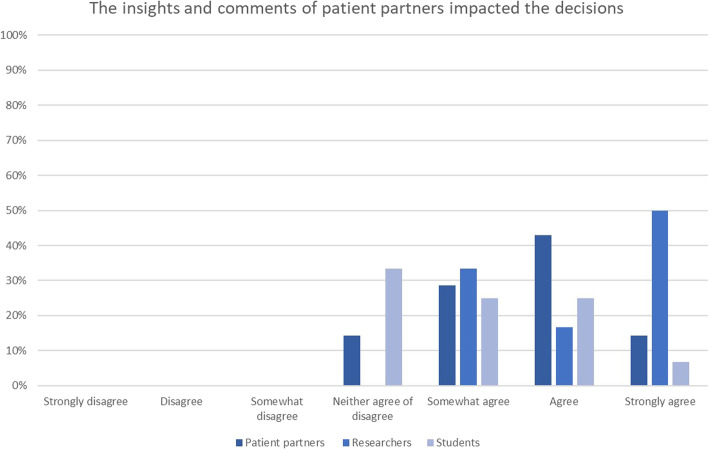
Responses to the statement ‘The insights and comments of patient partners impacted the decisions’ shown per group (students, patient partners and researchers)

Figure 3 shows that most researchers and patient partners felt that the insights and comments of patient partners impacted the decisions. As one patient partner shared:

“*I feel that the researchers really valued the patient partners and genuinely felt it was a critical element to project success. Our input really helped to shape the grant proposal” (patient partner 2, project 7)*.

While another patient partner shared:

*“The information I was asked to review was of such a general nature that I couldn't really comment on it helpfully except in rare cases of confusing writing” (patient partner 1, project 6)*

A researcher shared:

*“Patient partners bring another perspective to the research study (whether it be the actual question, interpretation of the results), often making the research more relevant and meaningful” (researcher 1, project 1)*

Less students (*n* = 5) agreed or strongly agreed that patient partners impacted decisions about their thesis project.

#### Context factors

Two researchers felt that patient engagement was not valued within their organization and two patient partners felt it was more a tick-box exercise. One patient partner shared:

*“We instigated this partnership, we were actually waiting to find a researcher who matched our values and interests. I would suggest our experience is rare, as I've had lots of other "tick the box" research and engagement experiences” (patient partner 1, project 3)*

### Results of patient engagement in the evaluation study

In this second results section, we report on the evaluation process and results of patient engagement in our evaluation study. Table 5 provides an overview of identified positive and negative outcomes and impacts of engaging patients in the evaluation study. Below we briefly describe the main findings.

**Table 5:** Overview of the outcomes and impacts of patient engagement in this evaluation study

Positive outcomes and impacts of patient engagement	Negative outcomes and impacts (or challenges) of patient engagement
The evaluation study is conducted in areas patients’ value	Challenge for the evaluation team to select evaluation questions relevant to all stakeholder groups
Patients influence decision-making and dissemination of evaluation findings	Reduced efficiency, increased time and resources
Increased transparency, applicability and validation of the evaluation findings	Struggle to find relevant measurement tools, extra time needed to adapt exiting tools to local needs
Learning about the variety of perspectives on evaluation of patient engagement, questions and evaluation objectives (all)	Tension between the scientific rigour and practical relevance for the community (researchers)
Increased understanding of research and confidence to speak up in other research projects (patient partners)	
Increased motivation to become involved in future research and evaluation studies (patient partners)	
Learning about research and evaluation, capacity building (patient partners)	
Increased understanding of co-designing an evaluation study and confidence that patients can be good co-investigators (researchers)	
Increased motivation to evaluate patient engagement (researchers)	
Fun experience (all)	

#### Outcomes and impacts of patient engagement on the evaluation study

The evaluation team felt that the involvement of patients helped choose relevant evaluation questions, validated the evaluation findings, reinforced transparency and increased the applicability of the work. Researchers of the evaluation team experienced a tension between the scientific rigour and relevance of evaluation tools, which can be seen as a challenge of patient engagement in designing an evaluation study. A researcher mentioned:

*“I remember one of our discussions in this project about choosing a response scale. Some of the Patients Canada tools had a yes/no scale and I did not want to use them. I wanted to use a strongly agree to strongly disagree scale and there is a very good reason for that methodologically and statistically, but I worried that I was overstepping or forcing that research belief on my patient partners. I hope I did not come across that way.” (researcher 1, evaluation team)*

Through our co-design process, we learned that the researchers on the evaluation team brought expertise related to the scientific process, while patients brought expertise about the relevance and readability of the survey questions. The redesigned survey may not reflect the validity of the original survey, which is inevitable when adaptions are made to validated tools. However, the researchers of the team learned that it is important to be flexible in the choice of research methods and measures and to pay attention to who defines and designs the content of evaluation tools. This process requires extra time and attention to a variety of viewpoints on what, how and when to evaluate patient engagement. The result is a comprehensive survey that reflects local needs, while respecting scientific rigour. The extent to which flexible approaches can be used may depend on the context of evaluation. For example, an evaluation of research projects across different provinces or countries may require some consistency in measures, while allowing adding questions of importance to patients or a specific research project.

#### Outcomes and impacts of patient engagement on the people involved

The researchers mentioned that the questions of patients motivated them to start the evaluation study. In addition, the process increased their confidence in patient engagement and proved to them that patients can be good co-investigators. As one researcher put it:

*“I’m really proud of the work we’ve done together. It’s really novel and can contribute to the literature.” (researcher 1, evaluation team)*

Patient partners reported impacts such as learning about research and the process increased their motivation to become involved in future studies with a greater comfort. One patient partner mentioned:

*“I started to realize what some of the research process was and became a bit more open mouthed in other research projects.” (patient partner 2, evaluation team)*

#### Process and context factors

We learned that stakeholders’ questions and evaluation objectives vary. For example, patients wondered how their contribution to a particular project was valued, while students and researchers wondered how well they were doing patient-oriented research and how their patient partners felt about their contributions. NL SUPPORT’s aim was to provide feedback and transparency about patient-oriented research and to enhance the support infrastructure. The challenge for the evaluation team was to select relevant questions for all involved. Nonetheless, the process was experienced as quite positive by all team members. A patient partner mentioned the following about the process:

*“The way we went about it was democratic, we have put all the ingredients in there that needed to be there which I don’t think is done in other evaluations; with the patient representatives, the theorists and the evaluators all being part of the process.” (patient partner 3, evaluation team)*

Facilitating factors included trust and respect between the evaluation team members. Patient partners noted that the researchers on the evaluation team were very open to engage patients and they showed appreciation which made patients feel comfortable. The study was conducted in about two years from the start until dissemination of the findings. None of the team members worked full time on the project and resources were limited.

## Discussion

This study provides insights into the participative development of a patient engagement evaluation study, and its findings. By understanding what and how to best monitor and evaluate patient engagement, feedback can be provided to those involved to enhance their engagement practice. The importance of feedback has also been described by Mathie et al. [33]. However, there are several challenges that evaluators have to tackle when selecting and implementing evaluation methods [34]. Below we reflect on our evaluation study findings and the participatory process.

### Reflection on the evaluation study findings

This pilot evaluation study looked at the expectations and attitudes patient partners, researchers and students have about patient engagement in health research (Q1), supporting factors and barriers (Q2), impact of engagement on health research (Q3) and uptake of research (Q4).

In terms of expectations and attitudes, our findings show that most researchers, patient partners and students value patient engagement in health research. Interestingly, we did not observe any differences in outcomes between those whose primary reason to engage patients was because it was required by the funder and those who felt patients and caregivers would add value. Our results show that midway their research project, all participants (strongly) believe that patient engagement improves the quality and uptake of research, which suggests that the attitude of researchers towards patient engagement could change over time.

Looking at the impact on health research and uptake of research, It was reported that patient partners impacted research decisions, though their influence could be higher in particular in student-led projects. Patients had contributed mostly to the initial stages, in line with systematic reviews [35]. Notably, however, our findings reveal high intention for patients to contribute to analysing the data, dissemination and implementation of study findings. Future evaluation will confirm whether indeed this happens and could possibly report on the impact patient engagement has on bringing knowledge to practice. As the projects included in this pilot evaluation study were midway through their research, we cannot report on the impact on the uptake of research.

Students, researchers and patient partners experienced barriers during the engagement process. Our findings show that students felt less prepared and were less satisfied with their patient engagement experience than researchers and their patient partners. While several students received training, more support and better capacity building at the supervisor level in academic institutions is needed. Patient partners reported barriers related to the communication between researchers and patient partners. The timing of engagement was not clear to all patient partners. Researchers could improve the partnership by communicating more frequently during the research process. Other studies reported similar barriers related to communication [36]. A detailed plan, including an overview of tasks and decision-points, as well as regular feedback and project updates could facilitate successful communication between patient partners and researchers. The most commonly reported challenge from a researcher perspective was the time commitment, though most recognized this was part of the process and necessary.

Our results show that most patients and researchers felt prepared and worked together in various phases of the research process which can be seen as a facilitating factor. This outcome may be related to the support infrastructure and the training sessions offered by NL SUPPORT. In Canada, patients, students and researchers are trained together about patient engagement, whereas in other countries, only patients are trained or they are trained separately. Furthermore, training for health researchers is not available in many countries [37]. A study conducted in the Netherlands showed that although patients were trained, this did not lead to structured patient engagement in health research; it was unclear when and how they could best work together [36]. Co-learning can help to clarify expectations and facilitate team building [20, 38]. The role of the principal investigator in building relationships and facilitating meaningful engagement should not be underestimated. De Wit et al. also emphasized the vital role of investigators regarding facilitation and support of patient partners [37]. Research funders can play a role in building a support infrastructure and could make training a requirement for research funding.

### Refection on patient engagement in this study

Overall, this was very much a patient-driven evaluation study, as we focused on questions patients wanted to see answered and engaged three patients as co-investigators throughout the entire evaluation study. Patients contributed equally to all phases of the evaluation process. Our experience is that patients bring valuable knowledge for designing evaluation studies. The involvement of patients helped choose relevant evaluation questions, validated the evaluation findings, reinforced transparency and increased the applicability of the work, but does require sufficient time for patient engagement. Participatory evaluation may increase the relevance and usefulness of information, but it also raises issues such as who defines and designs the content of evaluation tools. The research enterprise is largely based upon the application of scientific methods, in which there is a strong focus on quantitative measures and validated methodologies (e.g., validated surveys) [39]. This does not leave much room to integrate experiential knowledge in a local context (e.g., adapting questions that are unclear or irrelevant from a patient perspective). We do not believe there was a trade-off between the scientific rigour and patient partner preferences in our evaluation design. In fact, we adapted the Patient Canada’s tool included yes/no response scale to a Likert scale, the latter generally preferable from a scientific rigour perspective, while we deleted and added questions relevant from a patient perspective. We suggest those who wish to evaluate patient engagement to first explore published tools as provided in the Patient and Public Evaluation Toolkit [29]. Depending on the evaluation context, questions and intended use of results, an evaluation tool may be found that is fit for purpose with perhaps some adaptations, if not new tools may need to be developed. We suggest that more flexible approaches to research methodologies are needed for the monitoring and evaluation of patient engagement, looking not only at validity from the perspective of researchers, but also from the perspective of patients. This has also been noted by Baines and Donovan [40]. Patient engagement should perhaps be defined as ‘meaningful and active collaboration in governance, priority setting, conducting research, knowledge translation and evaluation’.

### Strengths and limitations

We captured the perspectives of students, researchers and patient partners which we see as a strength. In particular, very little literature has investigated student perceptions of patient engagement, but this is important for the next generation of researchers. A limitation of our study is the small number of participants. It should also be noted that the patient participants may not be representative of the general population (e.g., mostly highly educated females) which may limit the generalizations of our findings. In addition, selection bias may have occurred as principal investigators were asked to select patient partners. Also, participation was voluntary, those who were less involved or had a negative experience may have decided not to participate in this study. Future studies may also wish to pay attention to the method or level of engagement, material and financial resources (e.g. compensation of patient partners) and if schedules were accommodated with work schedules, as this may influence experiences and outcomes. Our evaluation study focused mainly on the impact of patient engagement on research decision-making. Though this was considered as important by patients and researchers, personal benefits such as increased confidence, less social isolation, new use of existing skills should not be overlooked, as well as negative impacts on peoples’ lives such as stress, feeling overwhelmed etc. [10, 41]

We created a comprehensive survey that reflects local needs, while respecting scientific rigour which we see as a strength. Using quantitative methods allowed us to easily collect information from participants with the opportunity to compare responses across groups, while complementing the data with more qualitative in-depth information about participants’ experiences. We found that the quantitative data had little variability – most participants rated the maximum on the Likert scale questions (e.g., a ceiling effect might have been reached). The selected projects largely differed (e.g., topics, engagement methods, types of research, people involved), therefore it is very difficult to evaluate patient engagement following traditional (statistical) methods and reinforces the need for flexibility in evaluation approaches. Interestingly, self-administered questionnaires and surveys were the most common type of tool identified [18]. Patient-oriented research is about the relational work between patients and researchers. The relational work is extremely difficult to evaluate but in many ways the most important as also emphasized by Abelson et al. [42]. The qualitative data provided valuable insights into peoples’ experiences. It is important to follow-up with focus groups or interviews to better understand the experiences and quantitative data. More innovative evaluation tools could also be used for this type of evaluation studies, e.g., quizzes, postcards, photo survey, video diaries. Mixed methods and multiple tools are needed to evaluate patient engagement, as one survey does not capture the complexity and outcomes of engagement. One of the difficulties is that the context in which engagement takes place often influences outcomes [43], and should therefore not be overlooked in evaluation studies.

We purposely chose internal evaluators, as our aim was to create a learning environment by involving relevant stakeholders in the evaluation process. Our approach was theory and stakeholder-based which we see as a strength. We agree with others that evaluators of processes like patient engagement should use mixed methods: assessing the process, its outcomes, and its context; taking into account both the theory and participants’ views; and being scientifically rigorous while also adaptive to local needs [34]. However, the lack of organizational capacity and resources needed for these types of evaluation studies should not be overlooked, as well as the time it takes before outcomes become visible. A co-creation process is required to develop appropriate monitoring and evaluation strategies. Evaluators should be aware that their position can influence an evaluation study; therefore, they should be transparent in how they select and implement monitoring and evaluation methods.

## Conclusion

Our findings show that researchers, patient partners and students value patient engagement in health research. We found that currently available tools for monitoring and evaluation do not fully capture local needs and the complexity of patient engagement. We argue that there is a need for participatory and flexible evaluation approaches in which all involved in patient engagement practices decide what to measure and how to measure it.

## Supplementary information

**Additional file 1.** GRIPP2 – Long form.

**Additional file 2.** Stakeholder engagement.

**Additional file 3.** Review grid evaluation tools.

**Additional file 4.** Patient partner - mid project survey.

**Additional file 5.** Researcher - mid project survey.

**Additional file 6.** Student survey.

## Data Availability

Data generated or analyzed during this study are included in this published article and its supplementary information files.
