# Supplementary material for: Giving patients a voice: a participatory evaluation of patient engagement in Newfoundland and Labrador Health Research
Source: Res Involv Engagem. 2020 Jul 9;6:39. doi: 10.1186/s40900-020-00206-5 (PMC7350650; doi:10.1186/s40900-020-00206-5)
Supplement: Supplementary file 1 — Additional file 1. GRIPP2 – Long form. [file 40900_2020_206_MOESM1_ESM.docx]

**Additional file 1: GRIPP2 – Long form**

| Section and topic | Item | Reported on page No |
| --- | --- | --- |
| Section 1: Abstract of paper | | |
| 1a: Aim | Our pilot evaluation study aimed to assess patient engagement in research projects, but at the same time provided insights into methods used for evaluation. This paper presents the results of our pilot evaluation study and our lessons learned regarding evaluation itself. | 1 |
| 1b: Methods | Patients were involved as co-investigators in this evaluation study | 1 |
| 1c: Results | Involvement of patients in our evaluation study resulted in learnings, transparency, validation of findings and increased applicability. A challenge was to select evaluation questions relevant to all involved and to adapt evaluation tools to local needs. | 1 |
| 1d: Conclusions | Participatory evaluation may increase the relevance and usefulness of information, but it also raises issues such as who defines and designs the content of evaluation tools. A co-creation process is required to develop appropriate monitoring and evaluation strategies. | 1 |
| 1e: Keywords | Patient engagement; patient and public involvement; patient participation; monitoring; evaluation; outcomes; participatory research; participatory evaluation | 1 |
| Section 2: Background to paper | | |
| 2a: Definition | There is no commonly accepted definition of ‘patient engagement’ and terms such as ‘patient and public involvement’ or ‘patient participation’ are also used. We used the Canadian Strategy for Patient Oriented Research (SPOR) definition of engagement for this study. Patient engagement is defined as ‘meaningful and active collaboration in governance, priority setting, conducting research and knowledge translation’. Patients contributing to research are known as ‘patient partners’, an overarching term for individuals with personal experience of a health issue and informal caregivers, including family and friends (CHIR, 2014). | 3 |
| 2b: Theoretical underpinnings | As our primary aim was to enhance patient engagement in health research, we decided to extend engagement in research to also evaluation; creating learning environment by involving relevant stakeholders in the evaluation process. Also known as participatory evaluation. | 4 |
| 2c: Concepts and theory development | Concepts and theory about participatory evaluation, stages and levels of engagement | 4 |
| Section 3: Aims of paper | | |
| 3: Aim | Our aim was to evaluate questions relevant to patients and to report results back to them to enhance their partnership. In addition, we aimed at learning more about how partnerships can be best supported and methods that can be used for evaluating patient engagement. | 3 |
| Section 4: Methods of paper | | |
| 4a: Design | Methods included: a participatory workshop with the patient advisory council  Three members of the advisory council joined the evaluation team (BD, MW, SG). They were involved in selecting the evaluation questions, the development of the measurement tools, piloting the survey, the interpretation of study findings and dissemination activities. | 9 |
| 4b: People involved | The council was established in 2014 and comprises over 20 residents from around the province with varying employment, training and health backgrounds. In addition, we engaged other key stakeholders such as the funding and the implementing agency (NL SUPPORT), researchers and an evaluation expert (NP). The team consisted of a principal investigator (LEV) and a co-principal investigator (HE). | 3, 9 |
| 4c: Stages of involvement | Additional file 2 provides an overview of the stakeholders involved per phase of the study |  |
| 4d: Level or nature of involvement | Additional file 2 provides an overview of the level of involvement per phase of the study |  |
| Section 5: Capture or measurement of PPI impact | | |
| 5a: Qualitative evidence of impact | We have used qualitative research methods such as observations, notes, recordings of meetings and written feedback to track patient inputs and our decision-making process. At the end of the study, we organized an online focus group with the evaluation team to reflect on our process and the impact patient engagement had on our evaluation study. | 9 |
| 5b: Quantitative evidence of impact | No quantitative methods have been used to evaluate the impact of patient engagement in our evaluation study, however we have used quantitative methods to evaluate patient engagement in the health research studies included in our evaluation study | 9 |
| 5c: Robustness of measure | In the discussion we comment on the method used to capture or measure the impact of patient engagement in the health research studies included in our evaluation study | 17 |
| Section 6: Economic assessment | | |
| 6: Economic assessment | No economic assessment has been conducted, however human resources (time, knowledge) have been measured in our study | NA |
| Section 7: Study results | | |
| 7a: Outcomes of PPI | An overview of positive and negative outcomes of patient engagement in our evaluation study is provided in table 2 and explained in the main text. | 14, 15 |
| 7b: Impacts of PPI | An overview of positive and negative impacts is provided in the results section and explained in the main text | 14, 15 |
| 7c: Context of PPI | The context is in-depth described on page 3 | 3 |
| 7d: Process of PPI | Factors that facilitated and inhibit the process of patient engagement in our evaluation study are described on page 8. Facilitating factors include trust, respect, knowing each other very well, early engagement. Inhibiting factors included extra time to conduct the study and the need to adapt existing tools to local evaluation questions. | 14, 15 |
| 7ei: Theory development | No specific conceptual or theoretical development has emerged, however we comment on our main lessons learned and reflect on this a broader context in the discussion. | 17 |
| 7eii: Theory development | We have not evaluated a specific theoretical model, however we have used the SPOR value model to categorize and report our findings. | 8 |
| 7f: Measurement | We have not tested the instruments used in our evaluation study on eg, validity, reliability, feasibility, acceptability, responsiveness, interpretability, appropriateness, precision. However, we conducted a small usability check before sending out the survey and we build on existing tools that had a high assessment score. | 14, 15 |
| 7 g: Economic assessment | We did not conduct and economic assessment | NA |
| Section 8: Discussion and conclusions | | |
| 8a: Outcomes | We feel that this was a very much patient-driven evaluation study. Patients influenced almost all phases of the study. Benefits include transparency, applicability to our province and validation of evaluation findings. Negative outcomes include the need to adapt existing evaluation tools and increased time. | 16, 17 |
| 8b: Impacts | Our paper describes the impact of patient engagement in evaluation as well as the impact of patient engagement in health research studies. | 16, 17 |
| 8c: Definition | We suggest to add ‘evaluation’ to the definition of patient engagement: ‘meaningful and active collaboration in governance, priority setting, conducting research, knowledge translation and evaluation’. | 17 |
| 8d: Theoretical underpinnings | Our study adds to the field of the science of engagement as well as the science of evaluating patient engagement. We suggest that more flexible approaches to research methodologies are needed for monitoring and evaluation of patient engagement, looking not only at validity from the perspective of researchers, but also from the perspective of patients. | 17 |
| 8e: Context | Greater attention to context factors could perhaps have improved our evaluation approach. All projects were funded by NL SUPPORT, who was also involved in the evaluation study. We had chosen for internal evaluators as our aim was to create a learning environment, by involving relevant stakeholders in the evaluation process. | 17, 18 |
| 8f: Process | We commented on facilitating and inhibiting factors in the results section and briefly reflected on our engagement process in the discussion. | 17 |
| 8 g: Measurement and capture of PPI impact | In our evaluation study, quantitative methods allowed us to easily collect information from participants with the opportunity to compare groups, while complementing the data with more qualitative in-depth information about participants’ experiences  Other qualitative methods such as focus groups or interviews could have been used. Mixed methods and multiple tools are needed to evaluate patient engagement, as one survey may not capture the complexity and outcomes of engagement. | 16, 17 |
| 8 h: Economic assessment | We have not conducted an economic assessment. We suggest that besides financial resources also human and material resources should be considered when doing an economic assessment. | NA |
| 8i: Reflections/critical perspective | We provided a recommendations to enhance communication between patients and researchers and also some suggestions for capacity building in the discussion. | 16, 17 |
