# Supplementary material for: Giving patients a voice: a participatory evaluation of patient engagement in Newfoundland and Labrador Health Research
Source: Res Involv Engagem. 2020 Jul 9;6:39. doi: 10.1186/s40900-020-00206-5 (PMC7350650; doi:10.1186/s40900-020-00206-5)
Supplement: Supplementary file 2 — Additional file 2. Stakeholder engagement. [file 40900_2020_206_MOESM2_ESM.docx]

**Additional file 2: Stakeholder engagement**

| **Research stage** | **Stakeholders involved** | **Level of engagement** | **Method of engagement** | **Final decision** |
| --- | --- | --- | --- | --- |
| Topic selection, formulating the study questions | Patient advisory council | Empowerment | Participatory workshop | Evaluation team |
| Identifying outcomes and measurements | Evaluation team | Collaboration | Focused meetings | Evaluation team |
| Development of the proposal (including the survey) | Evaluation team, NL SUPPORT directors | Collaboration | Focused meetings and digital feedback | NL SUPPORT and ethics board |
| Designing of recruitment and consent procedures | Evaluation team and ethics advisor | Consultation | Focused meetings | Ethics board |
| Data collection and analysis | Study participants | Consultation | Survey | Investigator team |
| Interpretation of findings | Evaluation team | Collaboration | Focused online meeting | Evaluation team |
| Dissemination plan | Evaluation team | Consultation | Focused online conversations | Evaluation team |
|  | NL SUPPORT | Consultation | Focused conversations |  |
| Dissemination | Evaluation team | Collaboration | Co-present, co-authors | Evaluation team |
