# Supplementary material for: Giving patients a voice: a participatory evaluation of patient engagement in Newfoundland and Labrador Health Research
Source: Res Involv Engagem. 2020 Jul 9;6:39. doi: 10.1186/s40900-020-00206-5 (PMC7350650; doi:10.1186/s40900-020-00206-5)
Supplement: Supplementary file 3 — Additional file 3. Review grid evaluation tools. [file 40900_2020_206_MOESM3_ESM.docx]

| **Tool** | **Developers** | **Health research focus** | **Focus of evaluation** | **Questions for patients** | **Questions for project lead/researchers** | **Expectations/**  **Attitudes (inputs)** | **Supporting factors and barriers**  **(process)** | **Meaningful impact on research**  **(outcomes)** | **Influence on uptake of research**  **(outcomes)** | **Tracking over-time** | **Evaluation method** |
| --- | --- | --- | --- | --- | --- | --- | --- | --- | --- | --- | --- |
| Public and Patient Engagement Tool (PPEET) | The PPEET is the product of a Canadian collaboration of researchers and public and patient engagement (PPE) practitioners led by McMaster University. | No | Focus on one engagement activity | Yes | Yes  Different questions for patients and researchers | No | Yes | Yes | Partly, only project lead perspective | No | Mixed method: quantitative (5-point Likert scale) and open-ended questions in a  survey (validated) |
| Research with Patients and Public involvement (RAPPORT) | Wilson et al. and the University of Hertfordshire Public Involvement in Research Group | Yes | Focus on a project in which patients are involved | Yes | Yes  Similar questions for patients and researchers | Yes | Yes | Yes | Yes | Possibly | Mainly qualitative method  Survey for project leads  Interviews patients, researchers and funders/networks  Log-sheet for patient partners |
| Patients as Partner in Research | Alies Maybee, Brian Clark, Annette McKinnon, Emily Nicholas Angl. | Yes | Focus on a project in which patients are part of the team/co-applicants. | Yes | Yes  Similar questions for patients and researchers | Partly | Yes | Yes | Yes, spreading of knowledge (end-project survey) | Yes | Mixed method:  Quantitative (7-point scale, yes/no) and open questions  Survey start, mid-project and end-project |

*See Patient and Public Evaluation Toolkit for assessment scores on scientific rigour, patient and public involvement in their creation, comprehensiveness and usability:* [*https://ceppp.ca/en/collaborations/evaluation-toolkit/#research*](https://ceppp.ca/en/collaborations/evaluation-toolkit/#research)
