# Supplementary material for: Giving patients a voice: a participatory evaluation of patient engagement in Newfoundland and Labrador Health Research
Source: Res Involv Engagem. 2020 Jul 9;6:39. doi: 10.1186/s40900-020-00206-5 (PMC7350650; doi:10.1186/s40900-020-00206-5)
Supplement: Supplementary file 4 — Additional file 4. Patient partner - mid project survey. [file 40900_2020_206_MOESM4_ESM.pdf]

Patient partner - Mid Project Survey

NL SUPPORT Unit - Evaluation team

Lidewij Eva Vat
Holly Etchegary
Nicole Porter
Mike Warren
Bud Davidge
Susan Goold

2017

Purpose

The survey is designed to understand the experiences of patients/caregivers when they partner with researchers/clinicians on a research project where the patients and/or caregivers are involved as a partner (not as a research subject).

Many of these items are taken directly or slightly adapted from Patients Canada Evaluation Tools. (see https://ossu.ca/for-patients/resources/ for the full evaluation tool). The questions were developed by patient/caregiver partners on research teams and represent areas they identified as important aspects of patients' experience with researchers.

Other items were adapted from: ReseArch with Patient and Public invOlvement: a RealisT evaluation – the RAPPORT study (https://www.ncbi.nlm.nih.gov/pubmedhealth/PMH0081028/).

Description

There are two draft surveys. The two surveys will allow for the tracking of the relationship over the duration of the project.

Mid Project - to be delivered about half-way through the research project
End Project - to be delivered after the research is completed and while it is in the KT phase

Acknowledgements

We would like to acknowledge the developers of the Patients Canada Evaluation Tools Alies Maybee, Brian Clark, Annette McKinnon, Emily Nicholas Angl and their reviewers Julia Abelson, PhD, Professor, Department of Clinical Epidemiology & Biostatistics, McMaster University, and Antoine Boivin, MD, PhD, Canada Research Chair in Patient and Public Partnership, Université de Montréal.

Questions for Patient Partners – mid project

Help us to understand your experience of partnering with researchers so we can learn what patient partners and researchers could use for support in future. Please read the information below carefully.

- With "Patient" we mean individuals with personal experience of a health issue and informal caregivers, including family and friends.
- This is a first survey of two surveys we are asking you to complete. You will receive another survey near the end of the project.
- Please answer the questions according to your experiences. There is no right or wrong answer.
- Please provide additional feedback in the comment boxes.
- All information you provide will remain confidential.
- The estimated time to complete this survey is about 10 minutes.

Thank you very much for your participation!

A Bit About the Project

1.	In which stage is the project to date?	☐ Planning research<br>☐ Doing research<br>☐ Sharing findings<br>☐ Project completed
2.	Including you, how many patients are partners are on this project at this time?	• ________________

Defining and Understanding Roles

3.	Did you and the Principal Investigator and/or team discuss your role?	Y/N
4.	Were the roles of the other members of the team explained to you?	Y/N
	Comments	

5.	Did <u>or</u> will you participate and contribute to the following: (Pick all that apply)<br><br>*(Pick all that apply)*	☐ Identifying & prioritizing topics (e.g. sharing problems and needs important to patients)<br><br>☐ Informing the design (e.g. assist in developing research questions, appropriate methods, recruitment strategy)<br><br>☐ Development of the grant proposal (e.g. writing or reviewing parts/all of the proposal)<br><br>☐ Managing (e.g. assist in writing patient information, consent forms, ethics application)<br><br>☐ Undertaking (e.g. assist in conducting interviews, surveys, focus groups)<br><br>☐ Analyzing & interpreting (e.g. assist in developing themes from data, interpret data)<br><br>☐ Dissemination (e.g. help distribute results, produce summaries, advise on channels for dissemination)<br><br>☐ Implementation (e.g. assist in developing patient information for new services/interventions)<br><br>☐ Monitoring & evaluation (e.g. continued involvement, help address issues, reflect on research process and roles)<br><br>☐ Other (please specify)

PARTICIPATING ON THE PROJECT

For each of the following statements, please indicate whether you: strongly disagree (1), disagree (2), somewhat disagree (3), neither agree or disagree (4), somewhat agree (5), agree (6), strongly agree (7).

6.	I am comfortable with my understanding of the research project.	(*Use a 7 point scale*)<br>(1) strongly disagree - (7) strongly agree
7.	I am comfortable speaking up and contributing during meetings	(*Use a 7 point scale*)<br>(1) strongly disagree - (7) strongly agree
8.	The research team listened to and absorbed my input.	(*Use a 7 point scale*)<br>(1) strongly disagree - (7) strongly agree
9.	How many hours have you spent working on the research project to date (on average)?	☐ Less than 1 hour a month<br>☐ 1 - 2 hours a month<br>☐ 3 - 4 hours a month<br>☐ More than 4 hours a month
10.	Have you worked as a patient partner on a research project prior to this one?	Y/N
11.	Have you attended any training sessions to help you in your role as a patient partner in health research?	Y/N
12.	What do you find difficult when helping the researchers/being involved?	

YOUR OVERALL ASSESSMENT

For each of the following statements, please indicate whether you: strongly disagree (1), disagree (2), somewhat disagree (3), neither agree or disagree (4), somewhat agree (5), agree (6), strongly agree (7).

13.	My insights and comments impacted the decisions of the team	(*Use a 7 point scale*)<br>(1) strongly disagree - (7) strongly agree
14.	I feel equipped to contribute to the research project.	(*Use a 7 point scale*)<br>(1) strongly disagree - (7) strongly agree
15.	I feel that the research team is well prepared to work with patient partners	(*Use a 7 point scale*)<br>(1) strongly disagree - (7) strongly agree
16.	The engagement experience is a good use of my time.	(*Use a 7 point scale*)<br>(1) strongly disagree - (7) strongly agree
17.	So far, I am satisfied with my experience as a patient partner on the research project.	(*Use a 7 point scale*)<br>(1) strongly disagree - (7) strongly agree

18.	I believe that patient partners can improve the quality and outcomes of research.	(*Use a 7 point scale*)<br>(1) strongly disagree - (7) strongly agree
19.	I think that patient partners can help with the translation and uptake of research.	(*Use a 7 point scale*)<br>(1) strongly disagree - (7) strongly agree
20.	What 3 things have you learned from the experience of partnering with researchers?	*____________________<br>*____________________<br>*_____
21.	What 3 things could the research team do to improve your experience?	*____________________<br>*____________________<br>*_____
22.	Based on your experience to date, do you think patient engagement in research is really supported or is more of a tick-box exercise?	☐ Really supported<br>☐ More a tick-box exercise
	Please comment on why you feel patient engagement is supported or more a tick-box exercise.	

A Bit About Yourself

23.	What year were you born?	
24.	What is your sex?	☐ Male<br>☐ Female<br>☐ Other
25.	Please describe the highest level of education you have completed:<br><br>*(Select one)*	☐ Less than high school<br>☐ High school<br>☐ Trade school or college<br>☐ University degree<br>☐ Other
26.	Please tell us your current work status<br><br>*(Select one)*	☐ Working part-time<br>☐ Working full-time<br>☐ Not in labour force, able to work<br>☐ Not in labour force, unable to work<br>☐ Retired<br>☐ Student (including students working part-time<br>☐ Homemaker
27.	You have experience with the healthcare system as:<br>*(Pick all that apply)*	☐ A patient<br>☐ A caregiver<br>☐ Paid healthcare professional in any capacity<br>☐ Academic health researcher<br>☐ Other (please specify)
28.	Please add any comments that you think may have been missed through the questions and/or that would assist in the evaluation	

Thank you for taking the time to provide your experiences and thoughts.
