# Supplementary material for: Giving patients a voice: a participatory evaluation of patient engagement in Newfoundland and Labrador Health Research
Source: Res Involv Engagem. 2020 Jul 9;6:39. doi: 10.1186/s40900-020-00206-5 (PMC7350650; doi:10.1186/s40900-020-00206-5)
Supplement: Supplementary file 5 — Additional file 5. Researcher - mid project survey. [file 40900_2020_206_MOESM5_ESM.pdf]

NEWFOUNDLAND AND LABRADOR SUPPORT
Support for People and Patient Oriented Research and Trials

Researcher - Mid Project Survey

NL SUPPORT Unit - Evaluation team

Lidewij Eva Vat
Holly Etchegary
Nicole Porter
Mike Warren
Bud Davidge
Susan Goold

2017

Purpose

The survey is designed to understand the experiences of researchers when they partner with patients/caregivers on a research project where the patients and/or caregivers are involved as a partner (not as a research subject).

Many of these items are taken directly or slightly adapted from Patients Canada Evaluation Tools. (see https://ossu.ca/for-patients/resources/ for the full evaluation tool). The questions were developed by patient/caregiver partners on research teams and represent areas they identified as important aspects of patients' experience with researchers.

Other items were adapted from: ReseArch with Patient and Public invOlvement: a RealisT evaluation – the RAPPORT study (https://www.ncbi.nlm.nih.gov/pubmedhealth/PMH0081028/).

Description

There are two draft surveys. The two surveys will allow for the tracking of the relationship over the duration of the project.

Mid Project - to be delivered about half-way through the research project

End Project - to be delivered after the research is completed and while it is in the KT phase

Acknowledgements

We would like to acknowledge the developers of the Patients Canada Evaluation Tools Alies Maybee, Brian Clark, Annette McKinnon, Emily Nicholas Angl and their reviewers Julia Abelson, PhD, Professor, Department of Clinical Epidemiology & Biostatistics, McMaster University, and Antoine Boivin, MD, PhD, Canada Research Chair in Patient and Public Partnership, Université de Montréal.

Questions for Researchers Partnering with Patients – Mid-term project

Help us to understand your experience of partnering with patients so we can learn what patient partners and researchers could use for support in the future. Please read the information below carefully.

- With "Patient" we mean individuals with personal experience of a health issue and informal caregivers, including family and friends.
- This is a first survey of two surveys we are asking you to complete. You will receive another survey near the end of the project.
- Please answer the questions according to your experiences. There is no right or wrong answer.
- All information you provide will remain confidential.
- The estimated time to complete this survey is about 10 minutes.

Thank you very much for your participation!

A Bit About the Project

1.	In which stage is the project to date?	☐ Planning research<br>☐ Doing research<br>☐ Sharing findings<br>☐ Project completed
2.	How many patients are partners on this project at this time?	

3.	Did or will patient partners participate and contribute to the following:<br><br>*(Pick all that apply)*	☐ Identifying & prioritizing topics (e.g. sharing problems and needs important to patients)<br><br>☐ Informing the design (e.g. assist in developing research questions, appropriate methods, recruitment strategy)<br><br>☐ Development of the grant proposal (e.g. writing or reviewing parts/all of the proposal)<br><br>☐ Managing (e.g. assist in writing patient information, consent forms, ethics application)<br><br>☐ Undertaking (e.g. assist in conducting interviews, surveys, focus groups)<br><br>☐ Analyzing & interpreting (e.g. assist in developing themes from data, interpret data)<br><br>☐ Dissemination (e.g. help distribute results, produce summaries, advise on channels for dissemination)<br><br>☐ Implementation (e.g. assist in developing patient information for new services/interventions)<br><br>☐ Monitoring & evaluation (e.g. continued involvement, help address issues, reflect on research process and roles)<br><br>☐ Other (please specify)

For each of the following statements, please indicate whether you: strongly disagree (1), disagree (2), somewhat disagree (3), neither agree or disagree (4), somewhat agree (5), agree (6), strongly agree (7).

4.	The patient partners are comfortable with their understanding of the research project.	(*Use a 7 point scale*)<br>(1) strongly disagree - (7) strongly agree
5.	Patient partners were comfortable speaking up and contributing at most meetings.	(*Use a 7 point scale*)<br>(1) strongly disagree - (7) strongly agree
6.	The research team listened to and absorbed the input from the patient partners.	(*Use a 7 point scale*)<br>(1) strongly disagree - (7) strongly agree
7.	How many hours have you and your team spent on patient engagement related activities to date (on average)<br><br>(for example: finding patient partners, preparing patient engagement activities, email or telephone contact with patient partners etc)	☐ Less than 1 hour a month<br>☐ 1 - 2 hours a month<br>☐ 3 - 4 hours a month<br>☐ More than 4 hours a month
8.	Have you worked with a patient partner on a research project prior to this one?	Y/N
9.	Have you attended any training sessions about patient engagement in health research?	Y/N
10.	What do find difficult working with patient partners?	

YOUR OVERALL ASSESSMENT

For each of the following statements, please indicate whether you: strongly disagree (1), disagree (2), somewhat disagree (3), neither agree or disagree (4), somewhat agree (5), agree (6), strongly agree (7).

11.	The insights and comments of patient partners impacted the decisions of the team.	(*Use a 7 point scale*)<br>(1) strongly disagree - (7) strongly agree
12.	Our patient partners are equipped to contribute to the research project.	(*Use a 7 point scale*)<br>(1) strongly disagree - (7) strongly agree

13.	I feel that my team and I are well prepared to work with patient partners on the research project	(*Use a 7 point scale*)<br>(1) strongly disagree - (7) strongly agree
14.	Patient engagement is a good use of my time and resources.	(*Use a 7 point scale*)<br>(1) strongly disagree - (7) strongly agree
15.	So far, I am satisfied with my experience of patient engagement on the research project.	(*Use a 7 point scale*)<br>(1) strongly disagree - (7) strongly agree
16.	I believe that patient partners can improve the quality and outcomes of research.	(*Use a 7 point scale*)<br>(1) strongly disagree - (7) strongly agree
17.	I think that patient partners can help with the translation and uptake of research.	(*Use a 7 point scale*)<br>(1) strongly disagree - (7) strongly agree
18.	What 3 things have you learned from the experience of partnering with patients?	*____________________<br>*____________________<br>*_____
19.	What 3 things could the patient partners do to improve your experience?	*____________________<br>*____________________<br>*_____
20.	Do you feel that patient engagement is valued within your organisation?	Y/N
	Please comment on how patient engagement is valued within your organization and/or what you feel should be changed.	

A BIT ABOUT YOURSELF

Please tell us a bit about who you are

21.	Where are you in your career as a researcher?<br>*(Select one)*	☐ Junior<br>☐ Mid-career<br>☐ Senior researcher
22.	What is your background?<br>*(Select one)*	☐ Clinician<br>☐ Researcher<br>☐ Clinician/researcher<br>☐ Other _____________
23.	What is your sex?	☐ Female<br>☐ Male<br>☐ Other
24.	What was the primary reason to have patient partners?<br>*(Select one)*	☐ Required by funder<br>☐ Felt patients and caregivers would add value<br>☐ Not my decision<br>☐ Other (please specify)
25.	Please add any comments that you think may have been missed through the questions and/or that would assist in the evaluation.	

Thank you for taking the time to provide your experiences and thoughts.
