# Supplementary material for: Giving patients a voice: a participatory evaluation of patient engagement in Newfoundland and Labrador Health Research
Source: Res Involv Engagem. 2020 Jul 9;6:39. doi: 10.1186/s40900-020-00206-5 (PMC7350650; doi:10.1186/s40900-020-00206-5)
Supplement: Supplementary file 6 — Additional file 6. Student survey. [file 40900_2020_206_MOESM6_ESM.pdf]

NEWFOUNDLAND AND LABRADOR
SUPPORT
Support for People and Patient Oriented Research and Trials

# Student - Survey

NL SUPPORT Unit - Evaluation team

Lidewij Eva Vat
Holly Etchegary
Nicole Porter
Mike Warren
Bud Davidge
Susan Goold

2017

## Purpose

The survey is designed to understand the experiences of students when they partner with patients/caregivers on a thesis project where the patients and/or caregivers are involved as a partner (not as a research subject).

Many of these items are adapted from Patients Canada Evaluation Tools. (see https://ossu.ca/for-patients/resources/ for the full evaluation tool). The questions were developed by patient/caregiver partners on research teams and represent areas they identified as important aspects of patients' experience with researchers.

Other items were adapted from: ReseArch with Patient and Public invOlvement: a RealisT evaluation – the RAPPORT study (https://www.ncbi.nlm.nih.gov/pubmedhealth/PMH0081028/).

## Acknowledgemens

We would like to acknowledge the developers of the Patients Canada Evaluation Tools Alies Maybee, Brian Clark, Annette McKinnon, Emily Nicholas Angl and their reviewers Julia Abelson, PhD, Professor, Department of Clinical Epidemiology & Biostatistics, McMaster University, and Antoine Boivin, MD, PhD, Canada Research Chair in Patient and Public Partnership, Université de Montréal.

**Questions for students**

Help us to understand your experience of partnering with patients so we can learn what patient partners and researchers could use for support in the future. Please read the information below carefully.

- With "Patient" we mean individuals with personal experience of a health issue and informal caregivers, including family and friends.
- Please answer the questions according to your experiences. There is no right or wrong answer.
- All information you provide will remain confidential.
- The estimated time to complete this survey is about 10 minutes.

Thank you very much for your participation!

1. In what year did your funding begin for your graduate work? (funding from TPMI/NL SUPPORT fellowship) _________

2. Has your thesis work started?

- Yes
- No

3. How did you find patient partners for your research team?

- Patient or caregiver organization
- Personal connections
- My supervisor(s) identified them
- Other ______________

4. Have you worked with a patient partner on a research project prior to this one?

- Yes
- No

5. Were the patient partners actively involved in discussing what you would do for your thesis project?

- Yes
- No

Please explain:

6. Did the patient partners participate and contribute to the following: *(Pick all that apply)*

☐ Identifying & prioritizing topics (e.g. sharing problems and needs important to patients)

- ☐ Informing the design (e.g. assist in developing research questions, appropriate methods, recruitment strategy)
- ☐ Development of the grant proposal (e.g. writing or reviewing parts/all of the proposal)
- ☐ Managing (e.g. assist in writing patient information, consent forms, ethics application)
- ☐ Undertaking (e.g. assist in conducting interviews, surveys, focus groups)
- ☐ Analyzing & interpreting (e.g. assist in developing themes from data, interpret data)
- ☐ Dissemination (e.g. help distribute results, produce summaries, advise on channels for dissemination)
- ☐ Implementation (e.g. assist in developing patient information for new services/interventions)
- ☐ Monitoring & evaluation (e.g. continued involvement, help address issues, reflect on research process and roles)
- ☐ Other (please specify)

7. Are there any of these areas where you feel patient partners can best contribute to a student thesis project? Please explain:

8. Please describe any patient engagement activities that have been used during your thesis project to date or those you have planned (e.g., consulted a patient advisory panel, invited patient partners to meetings about your project, asked for feedback on study instruments, etc.)

9. Have you attended any training sessions about patient engagement in health research?
   - Yes
   - No

10. Have you received any support to help you working with patients as partners?
    - Yes
    - No

Your overall assessment

For each of the following statements, please indicate whether you: strongly disagree (1), disagree (2), somewhat disagree (3), neither agree or disagree (4), somewhat agree (5), agree (6), strongly agree (7).

| | | |
|---|---|---|
| 11. | The insights and comments of patient partners impacted the decisions about my thesis | (*Use a 7 point scale*)<br>(1) strongly disagree - (7) strongly agree |
| 12. | The patient partners were equipped to contribute to the research project. | (*Use a 7 point scale*)<br>(1) strongly disagree - (7) strongly agree |

| | | |
|---|---|---|
| 13. | I feel that I was well prepared to work with patient partners on my thesis project | (*Use a 7 point scale*)<br>(1) strongly disagree - (7) strongly agree |
| 14. | I am satisfied with my experience of patient engagement on my thesis project. | (*Use a 7 point scale*)<br>(1) strongly disagree - (7) strongly agree |
| 15. | I have made changes in my thesis project because of what patient partners have said. | (*Use a 7 point scale*)<br>(1) strongly disagree - (7) strongly agree |
| 16. | I believe that patient partners can improve the quality and outcomes of research. | (*Use a 7 point scale*)<br>(1) strongly disagree - (7) strongly agree |
| 17. | I think that patient partners can help with the translation and uptake of research. | (*Use a 7 point scale*)<br>(1) strongly disagree - (7) strongly agree |
| 18. | Please share any examples of suggestions or comments patient partners made that helped you with your thesis project | |

A bit about yourself

| | | |
|---|---|---|
| 19. | Please name the faculty in which your thesis work is being conducted | |
| 20. | What is your sex? | • Female<br>• Male<br>• Other |
| 21. | What was the primary reason to have patient partners on your project?<br>*(Select one)* | • Required by funder<br>• Felt patients and caregivers would add value<br>• Not my decision<br>• Other |

| | | |
|---|---|---|
| 22. | Please add any comments that you think may have been missed through the questions and/or that would assist in the evaluation. | |

Thank you for taking the time to provide your experiences and thoughts.
